# Supplementary material for: Preparation and Characterization of Magnetic Fe3O4/CdWO4 and Fe3O4/CdWO4/PrVO4 Nanoparticles and Investigation of Their Photocatalytic and Anticancer Properties on PANC1 Cells
Source: Materials (Basel). 2019 Oct 8;12(19):3274. doi: 10.3390/ma12193274 (PMC6803879; doi:10.3390/ma12193274)
Supplement: Supplementary file 1 [file materials-12-03274-s001.pdf]

materials

MDPI

Article

Preparation and characterization of magnetic $Fe_3O_4/CdWO_4$ and $Fe_3O_4/CdWO_4/PrVO_4$ nanoparticles and investigation of their photocatalytic and anticancer properties on PANC1 cells

Mohammad Amin Marsooli [1,2], Mahdi Fasihi-Ramandi [1,2], Kourosh Adib [3], Saeid Pourmasoud [4], Farhad Ahmadi [5,6], Mohammad Reza Ganjali [7,8], Ali Sobhani Nasab [9,10], Mahdi Rahimi Nasrabadi [1,2,*] and Marta E. Plonska-Brzezinska [11,*]

1. Department; Nanobiotechnology Research Center, Baqiyatallah University of Medical Sciences, Tehran postcode, Iran; mohammadamin.marsuli@gmail.com.
2. Faculty of Pharmacy, Baqiyatallah University of Medical Sciences, Tehran postcode, Iran; fasihi.m@gmail.com.
3. Department of Chemistry, Imam Hossein University, Tehran postcode, Iran; k_anbaz@yahoo.com.
4. Department of Physics, University of Kashan, Kashan postcode, Iran; SA_POURMASOUD2007@yahoo.com.
5. Physiology Research Center, Iran University of Medical Sciences, Tehran postcode, Iran; Farhadahmadi55@gmail.com.
6. Department of Medicinal Chemistry, School of Pharmacy-International Campus, Iran University of Medical Sciences, Tehran postcode, Iran.
7. Center of Excellence in Electrochemistry, Faculty of Chemistry, University of Tehran, Tehran postcode, Iran; Ganjali@gmail.com.
8. Biosensor Research Centre, Endocrinology & Metabolism Molecular and Cellular Research Institute, Tehran University of Medical Sciences, Tehran postcode, Iran.
9. Social Determinants of Health (SDH) Research Center, Kashan University of Medical Sciences, Kashan postcode, Iran; Ali.sobhaninasab@gmail.com.
10. Core Research Lab, Kashan University of Medical Sciences, Kashan postcode, Iran.
11. Department of Organic Chemistry, Faculty of Pharmacy with the Division of Laboratory Medicine, Medical University of Bialystok, Mickiewicza 2A, 15-222 Bialystok, Poland; marta.plonska-brzezinska@umb.edu.pl.

* Correspondence: Rahiminasrabadi@gmail.com; Tel.: +98 2182483409 (M.R.N.); marta.plonska-brzezinska@umb.edu.pl (M.E.P-B.)

Received: 06 September 2019; Accepted: 04 October 2019; Published: date

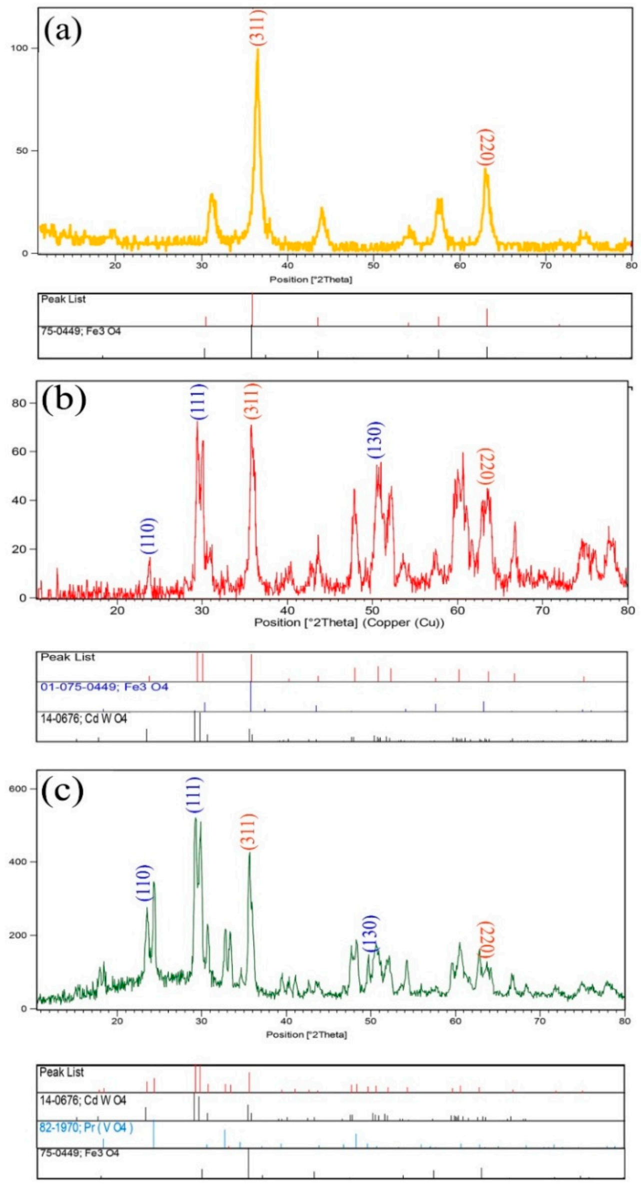

Figure S1. XRD patterns of (**a**) Fe_3O_4, (**b**) $Fe_3O_4/CdWO_4$ (S1), and (**c**) $Fe_3O_4/CdWO_4/PrVO_4$ (S4).

Table S1. Main reflections in XRD patterns of inorganic nanoparticles.

Sample	2 θ (degrees)	hkl	Crystallite sizes Dc (nm)	Average Crystallite size	A (A)	B (A)	C (A)
Fe_3O_4	35.6	(311)	14.58	14.1	8.3740	8.3740	8.3740
	63.1	(220)	13.71				
$Fe_3O_4/CdWO_4$	35.6	(311)	15.31	33.5	5.0290	5.8590	5.0740
	63.1	(220)	14.65				
	23.20	(110)	11.45				

	29.00	(111)	12.45				
	51.11	(130)	13.48				
**$Fe_3O_4/CdWO_4/PrVO_4$**	35.6	(311)	15.96	55	7.3990	7.3990	6.4960
	63.1	(220)	15.22				
	23.20	(110)	12.43				
	29.00	(111)	13.24				
	51.11	(130)	14.35				
	24.03	(200)	11.86				
	32.40	(112)	12.77				
	47.86	(322)	13.98				

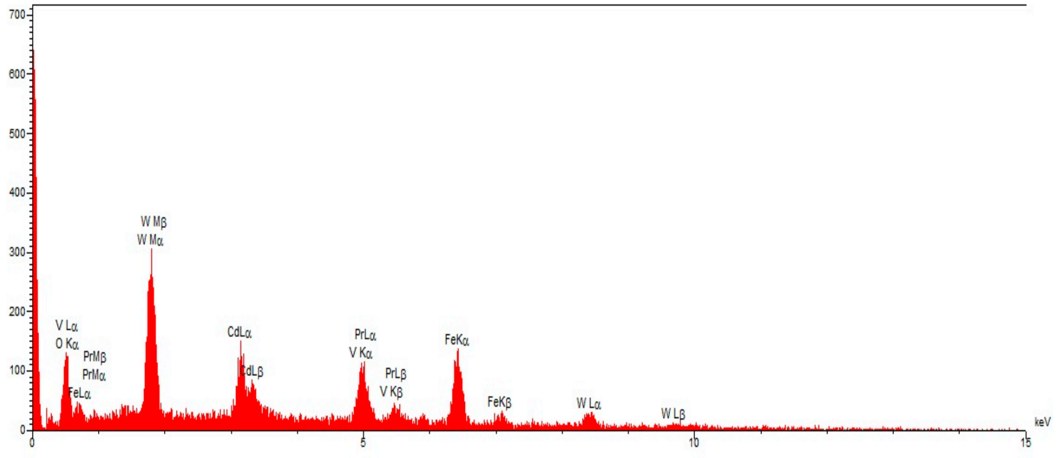

Figure S2. EDX spectrum of the $Fe_3O_4/CdWO_4/PrVO_4$ (S_4) sample.

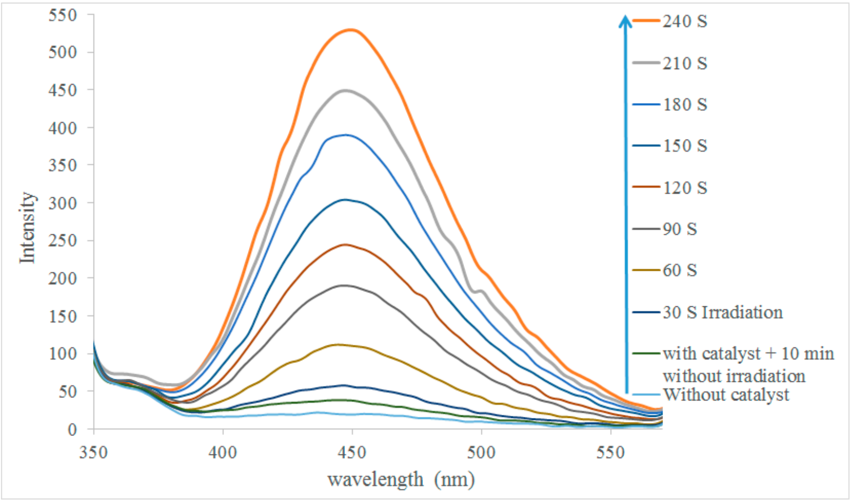

Figure S3. The temporal changes in fluorescence intensity under UV light (290 nm) irradiation for S4.

Table S2. The photoluminescence technique was used to study the generation of active •OH radicals for all sample (S1-S5).

Sample	Intensity (after 240 s of irradiation)
S1	350
S2	410
S3	485
S4	525
S5	500

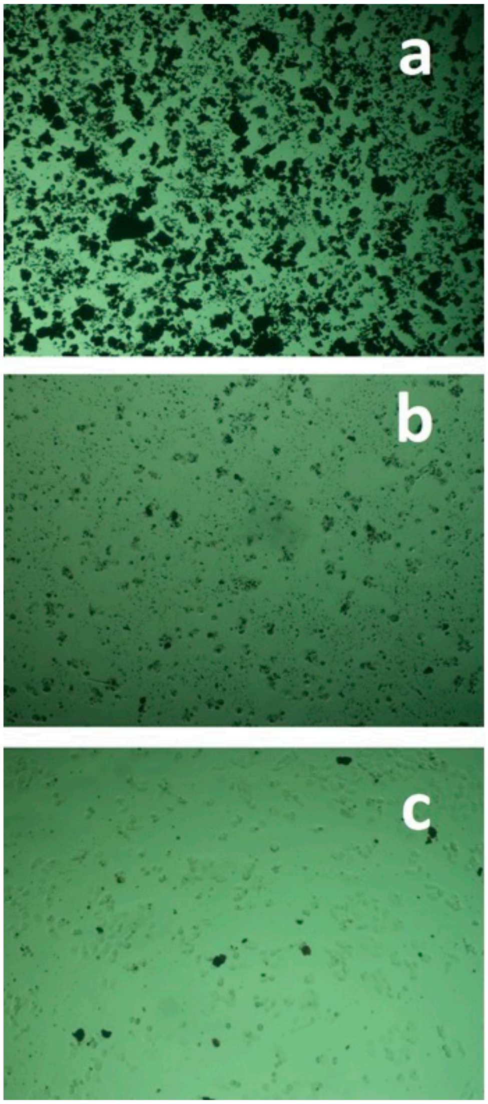

Figure S4. Microscopic photograph of PANC1 cell in the presence of S4 at three concentrations (**a**) 2, (**b**) 0.5, and (**c**) 0.0157 mg/mL.
